# Functional Recombinants Designed from a Fetuin/Asialofetuin-Specific Marine Algal Lectin, Rhodobindin

**DOI:** 10.3390/md13042183

**Published:** 2015-04-13

**Authors:** Jong Won Han, Min Gui Jung, Eun Young Shim, Jun Bo Shim, Young Min Kim, Gwang Hoon Kim

**Affiliations:** 1 Department of Biology, Kongju National University, Kongju 314-701, Korea; E-Mails: jwhan@kongju.ac.kr (J.W.H.); mgjung@kongju.ac.kr (M.G.J.); tlasud@kongju.ac.kr (E.Y.S.); matthew80@kongju.ac.kr (J.B.S.); newkym@kongju.ac.kr (Y.M.K.); 2 Marine Biodiversity Institute of Korea, Seocheon 427-100, Korea

**Keywords:** lectin, Rhodobindin, recombination, mass production, *E. coli* expression system

## Abstract

Plant lectins have attracted much attention for biomedical applications including targeted drug delivery system and therapy against tumors and microbial infections. The main problem of using lectins as a biomedical tool is a batch-to-batch variation in isoforms content. The production of lectins using recombination tools has the advantage of obtaining high amounts of proteins with more precise properties, but there are only a handful of functional recombinant lectins presently available. A fetuin/asialo-fetuin specific lectin, Rhodobindin, has unique tandem repeats structure which makes it useful in exploiting for recombinant lectin. We developed three functional recombinant lectins using *E. coli* expression system: one from full cDNA sequence and two from fragmentary sequences of Rhodobindin. Hemagglutinating activity and solubility of the recombinant lectins were highest at OD 0.7 cell concentration at 20 °C. The optimized process developed in this study was suitable for the quality-controlled production of high amounts of soluble recombinant lectins.

## 1. Introduction

Plant lectins have been exploited for biomedical diagnosis such as carbohydrate profiling of cell surfaces [1] and the isolation and characterization of glycoproteins [2]. Recently lectins have attracted attention for more direct biomedical applications including targeted drug delivery systems [3] and therapy against microbial infections or tumors [4,5], especially due to their ability to induce programmed cell death and/or autophagocytosis in cancer cells [6,7]. Quality control of the protein is essential to guarantee a constant result in medicinal application [8]. A lectin isolated from natural sources is not an option for medical use because most plants have a heterogeneous mixture of several lectin isoforms with distinct biological activities, which often cause “batch-to-batch” variation in isoform content. It is also very difficult to obtain large amounts of lectins with defined amino acid sequences and precise properties from natural sources. For this reason, the production of lectins by recombinant means is essential for successful medical application. Although a large number of lectins have been isolated and many of them are commercially available, there are only a handful of recombinant lectins which may be applicable for medical use at present [9].

Lectin-carbohydrate complementary systems has been proposed for gamete-gamete recognition and binding in red algae [10,11,12]. Recently the female-specific lectin involved in this process has been isolated from *Aglaothamnion callophyllidicola* and named Rhodobindin [13]. Interestingly tandem repeats was revealed in the cDNA sequence of this lectin, although the biological properties of individual repeat fragments have not been clarified [13]. If each fragment of the tandem repeat contains functional sugar-binding domain it could be possible to design several different recombinant proteins from the cDNA sequence. The subtle differences in amino acid sequence of each tandem repeat may be useful in studying the sugar-binding affinity of the lectin [13]. Rhodobindin may also have a strong potential as a drug delivery protein because of its specificity to fetuin and asialofetuin. Fetuins are blood proteins that are made in the liver and secreted into the bloodstream. They belong to a large group of carrier proteins mediating the transport of a wide variety of cargo substances in the bloodstream [3]. An asialofetuin-labeled liposomes was used for receptor-mediated transfer of specific DNA sequence to mouse liver cells almost two decades ago [14]. Recent studies showed that fetuin-A acts as an endogenous ligand to promote lipid-induced insulin resistance [15]. A recombinant lectin showing different binding affinity to fetuin/asialofetuin may be useful in designing drug delivery system.

In this study, we aimed to develop efficient expression system for functional recombinants of Rhodobindin. Characterization of active domains was conducted using recombinants designed from whole and fragmentary ORF (Open Reading Frame) sequence of the lectin.

## 2. Results

### 2.1. Cloning of Recombinant Lectins

**Figure 1:**
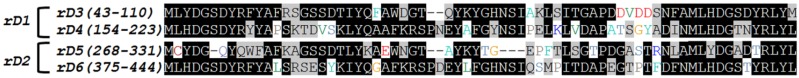
Multiple alignments of putative domains (rD3–rD6). Similarities between the sequences are shown with shaded background. Highly identical regions are marked with black bars Rhodobindin domain 1–6 (rD1–rD6) were cloned for expression of proteins. Deduced amino acid sequence and location of domains were shown at Figure S2.

Rhodobindin is a monomeric lectin with molecular size of 50.7 kDa. The protein consists of two heterogeneous domains (domain 1 and domain 2) which show 70.8% of similarity in amino acid sequence (Figure 1) [13]. Each domain consisted of four conserved regions which show high similarity to the corresponding region of the other domain (Figure 1). In order to know if the tandem repeats could make a functional lectin the full ORF of Rhodobindin was divided into six units (rD1–rD6), and cloned into expression vector together with full ORF of Rhodobindin (Figure 1, Table 1). The amino acids sequence of recombinant proteins were confirmed using MALDI-TOF mass spectrometry (Supplementary Figure S1).

**Table 1:** Primers used for construction of recombinant lectins.

Name	Sequence(5′-3′)	Sequences Amplified
Rhodo(1)-EcoRI-F1	GGATCCGAATTCATGTCTCGCTCATTCAAC	Whole, rD1, rD3 (forward)
Rhodo(228)-XhoI-R1	TCGGCGTCGCGCTGCCCCTCGAGCACCACC	rD1, rD4 (reverse)
Rhodo(229)-EcoRI-F1	GGATCCGAATTCACGCTGCGCCAGTTCGGC	rD2, rD5 (forward)
Rhodo(443)-XhoI-R1	CGATCCAGGCCGTTGCCCTCGAGCACCACC	Whole, rD2, rD6 (reverse)
Rhodo(116)-XhoI-R1	GCAAGCGCGCGACGGACCTCGAGCACCACC	rD3 (reverse)
Rhodo(113)-EcoRI-F1	GGATCCGAATTCCGCGCGACGGACGCGCTC	rD4 (forward)
Rhodo(337)-XhoI-R1	TGTCGCGCACGGGCGCGCTCGAGCACCACC	rD5 (reverse)
Rhodo(334)-EcoRI-F1	GGATCCGAATTCCGCACGGGCGCGTCGCTG	rD6 (forward)

### 2.2. Expression of Recombinant Rhodobindin

**Figure 2:**
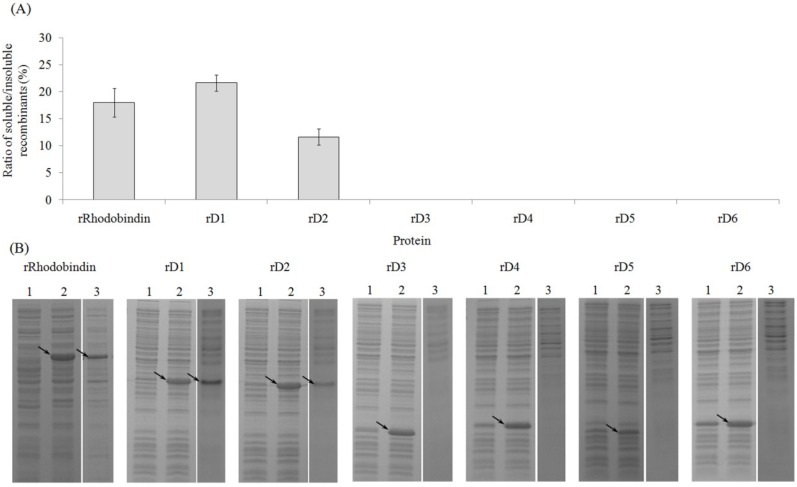
Expression of recombinant lectins at control condition (37 °C, OD 0.5, 0.5 mM IPTG induction for 2 h). (**A**) Ratio of soluble and insoluble fractions of different recombinants; (**B**) Gel electrophoresis of extracted total protein. Lane 1, bacterial protein; lane 2, inclusion bodies; lane 3, soluble fraction. Arrows indicate expressed recombinant proteins.

**Figure 3:**
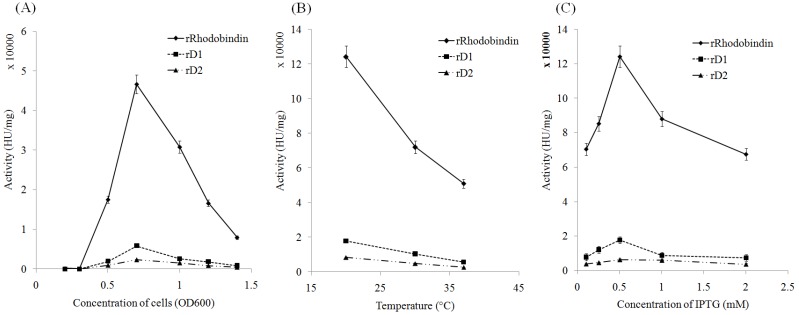
Expression efficiency of recombinant lectin in different conditions. (**A**) Changes of activity at different cell concentration (at 37 °C, 0.5 mM IPTG induction for 2 h); (**B**) Temperature effect on activity (OD600 = 0.7, 0.5 mM IPTG induction for 2 h) (**C**) Changes of activity at different IPTG concentration. (at 20 °C, OD600=0.7, for 2 h) (*n* = 3).

All recombinants (full and partial) were successfully expressed but only three recombinants: full ORF, domain 1 and 2 yielded soluble fractions (Figure 2). The soluble fraction from the full ORF and rD1 was about 20% of total fractions and the others yielded smaller soluble fractions (Figure 2). To optimize efficiency of soluble expression and hemagglutinating activities, different expression conditions were tested. Optimum cell concentration for expression of recombinant protein was at 0.7 OD. The hemagglutinating activity increased until the optical density reached 0.7 but began to decrease sharply at higher OD values (Figure 3a). Hemagglutinating activity of the recombinant lectin was also affected by temperature and IPTG (isopropyl-beta-d-thiogalactopyranoside) concentration. The activity at 20 °C was 2.5 fold higher than that at 37 °C (Figure 3b). The optimal concentration of IPTG for expression efficiency of recombinants was 0.5 mM at 20 °C (Figure 3c).

### 2.3. Purification of Recombinant Lectins

**Figure 4:**
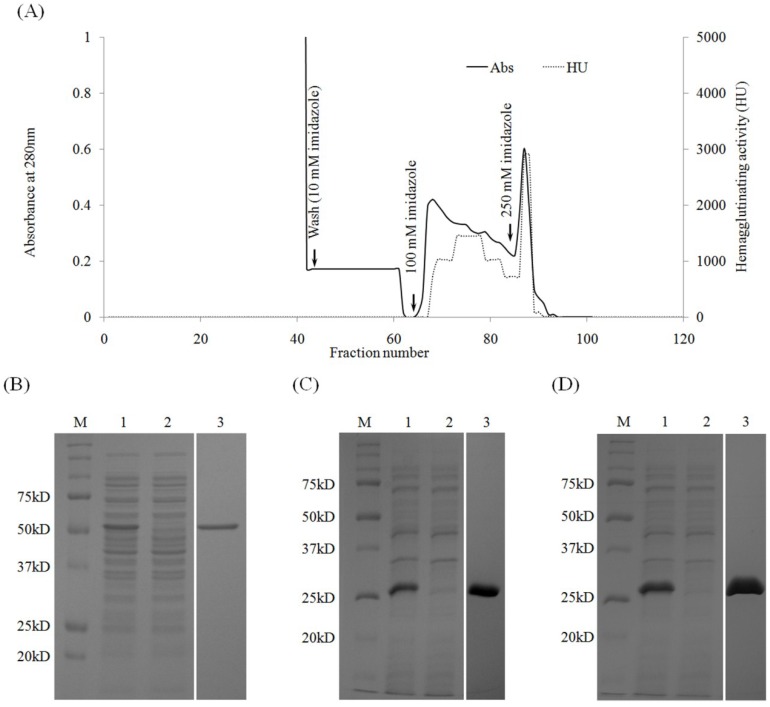
Purification of recombinant lectin using Ni-NTA agarose chromatography. (**A**) Chromatogram showing elution of protein from the column; (**B**–**D**) SDS-PAGE (Sodium dodecyl sulfate polyacrylamide gel electrophoresis). M, molecular weight marker; lane 1, crude extract; lane 2, flow-through fraction; Lane 3, purified recombinant lectin.

Ni-NTA agarose was used for purification of recombinant lectins. Most of recombinant lectins were bound to the affinity matrix. The lectins were purified by adding 100 mM of imidazole solution (Figure 4a). Remaining proteins in the column were eluted with higher concentration of imidazole. Only one band was observed from the eluted fraction of each recombinant (Figure 4b–d). After affinity chromatography, 80% of protein was recovered with purification increased 31-fold (Table 2). A maximum of 16.5 mg of active full ORF recombinant (rRhodobindin) was obtained from 1 L bacterial culture with a specific activity of approximately 4 × 10^6^ HU/mg of protein (Table 2 and Table 3). The recombinants from domain 1 and 2 (rD1 and rD2) showed similar expression patterns to that of rRhodobindin (Figure 2 and Figure 4c,d). However, hemagglutinating activity of the recombinants from domain 1 and 2 was about 15 (rD1)–30 (rD2) times lower than rRhodobindin. The mixture of rD1 and rD2 did not show increased activity (Table 3).

**Table 2:** Purification of rRhodobindin from 50 mL bacterial culture.

	Total Protein (mg)	Protein Concentration (mg·mL^−1^)	Total Activity (titer)	Specific Activity (titer·mg^−1^)	Purification Fold	Percentage of Recovery
Crude extract	26	0.65	3,276,800	126,030	1	100
Affinity chromatography	0.66	0.041	2,621,440	3,971,878	31	80

**Table 3:** Yield of recombinant proteins and Hemagglutinating activity.

Name	Molecular Weight (Kda) §	Yield (mg per 1L Culture)	Minimum Concentration (µg/mL) ¶	Minimum Mole Concentration
Full-rRhodobindin	50	16.5	0.04	0.8 nM
rD1	25	12.1	0.31	12.4 nM
rD2	25	13.3	0.63	25.2 nM
rD1 + rD2 (mixed) £	-	-	0.44	-

§: Based on amino acids sequence; ¶: Minimum concentration of proteins to aggregates 4% horse erythrocytes; £: Equal amounts of proteins were mixed.

### 2.4. Carbohydrate Specificity and Heat-Stability of Recombinant Lectins

**Figure 5:**
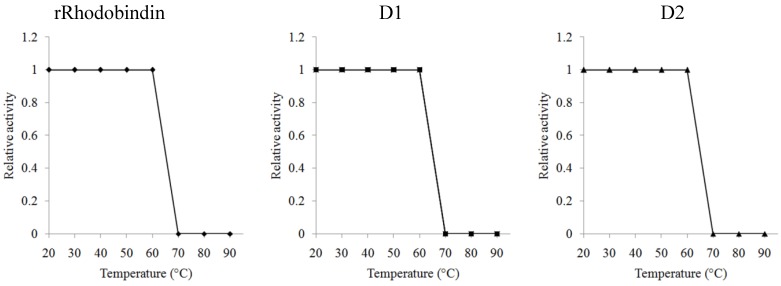
Heat stability of recombinant lectin activity (rRhodobindin, rD1, rD2) at various temperatures (20–90 °C). Relative activity measure to control at 20 °C.

The recombinant lectins did not require any divalent ion for their activities (data not shown). The sugar specificity of the three recombinant lectins was similar to native Rhodobindin (data not shown). Hemagglutinating activity of the lectins was not affected by any mono- or disaccharide up to 500 mM concentration but was completely blocked by fetuin (100 µg/mL) and asialo-fetuin (50 µg/mL). The three recombinant lectins were heat-stable (Figure 5). The activity was maintained until 60 °C but completely lost at 70 °C.

## 3. Discussion

Three functional recombinant lectins were designed using the cDNA information of a marine algal lectin Rhodobindin and produced in an *E. coli* expression system. rRhodobindin showed hemagglutinating activity as strong as that of the native lectin, but the recombinants from the fragmentary sequences (rD1 and rD2) showed weaker activity. Although rD1 and rD2 showed only 70.8% sequence similarity to each other they showed the same sugar specificity. Four tandem repeats of a conserved amino acid sequence (ML[H/Y]DG[S/A]D[Y/T]R[L/Y/F]Y) was present in each domain, which appears to contribute to the binding activity of the lectin. The soluble expression of recombinants was affected by temperature, cell density and IPTG concentration, but the optimized conditions for the production of lectin were almost the same for all recombinants. All recombinants were heat stable and did not require any divalent ions for hemagglutinating activity. These characteristics of recombinants may render them useful in medical application in which heat treatment is required.

Recombinant production of lectins in microbial hosts is an efficient way to overcome disadvantages of using lectins from their natural sources because it will ensure continuous supply and facilitate purification of lectins with tailor-made functionalities, particularly for biomedical application [16]. Although the *E. coli* expression system is a very productive and economic way to produce recombinant lectins, it can have difficulties in expression of glycoproteins and solubility of the product. Recombinant expression in bacterial system often results in the production of inclusion bodies instead of active soluble proteins, which could be due to unfolded or mis-folded proteins [17]. Two strategies for recovery of active soluble proteins are commonly used, which are refolding of proteins during purification steps and controlling expression level in bacterial system [17,18], but both require additional expensive steps. The matrix based refolding procedures has been popularly used in early recombinant studies but it is inefficient for the lectins applicable for biomedical use because it requires chromatography system and takes too long time for final production [17]. For our recombinant lectins, the optimization of culture and induction conditions was enough to generate active soluble protein.

Several eukaryotic hosts have been exploited to overcome problems of insoluble expression of the bacterial system and to produce glycosylated lectins but these systems also have problems which need to be considered; codon usage for optimization, strain culture, fusion partners, induction conditions, and purification methodology [19,20]. Rhodobindin has great potential to develop recombinant lectins for biomedical application because it is water soluble and has a secondary structure with tandem repeats of conserved peptide sequences. These characteristics make it easy to design several different forms of recombinants from an ORF sequence. Three soluble recombinants developed in this study showed high yields comparable to other recombinant lectins. Approximately 16.5 mg of rRhodobindin was produced from 1 L of bacterial culture, while the yield of lectin in other studies were 16 mg/L for frutalin [21], and 15 mg/L for *Microcystis viridis* lectin [22].

Rhodobindin is characterized by four conserved peptide tandem repeats in two domains, Shim *et al.* [13] suggested that these tandem repeats may be responsible for the lectin activity. Our results showed that recombinants of each domain, containing four tandem repeats, could make an active lectin supporting the suggestion. The three recombinants showed the same sugar specificity suggesting that the subtle difference of amino acid between the domains did not affect the sugar specificity. The hemagglutinating activity of rD1 and rD2 was almost the same but 15–30 times lower than rRhdobindin. It is possible that the strength of activity may be dependent on the number of tandem repeats in each recombinant. It has been shown that carbohydrate-binding affinity of recombinant lectins could be enhanced by an array of tandem repeats of the binding sites [23]. Further studies using crystallography is essential to understand how these tandem repeats contribute to sugar binding activity.

Rhodobindin shows no sequence homology with any reported proteins [13]. It is a bit surprising because lectins are one of the most thoroughly studied groups of proteins and at least several hundred lectins have been isolated and classified with their secondary structures in terrestrial plants and animals [24]. The secondary structure of Rhodobindin is similar to galectin from the nematode *Caenorhabditis elegans*. Galectin also has two repeat structures in *N*- and *C*-terminal lectin domains. Both domains have the same sugar specificity but the binding properties are different [25,26]. However, Galectin is different from Rhodobindin in not having tandem repeats of conserved peptide sequences. Shim *et al.* [13] suggested that the unique structure of Rhodobindin is due to the fact that the protein mediates gamete recognition.

As a consequence of their chemical properties and binding specificity, lectins have a strong potential as a tool for drug delivery [27,28]. The idea to use lectins for drug delivery came more than two decades ago. Woodley and Naisbett [29] proposed the use of tomato lectin (TL) to target the luminal surface of the small intestine for the first time. The lectin-sugar interaction has also been used to trigger vesicular transport into or across epithelial cells. Early studies revealed that some lectins can mediate mucoadhesion, cytoadhesion, and cytoinvasion of drugs [30]. A lectin-grafted pro-drugs and carrier systems have been exploited for decades to improve absorption and bioavailability of poorly absorbable drugs, peptides and proteins as well as therapeutic DNA [31].The concept of bio adhesion via lectins has been applied to detour biological barriers like the nasal mucosa, the lung, the buccal cavity, the eye and the blood-brain barrier [3]. Our recombinant lectins may be very useful in detouring biological barriers especially because they are fetuin/asialofetuin specific.

A lectin specific to fetuin/asialofetuin is particularly useful in drug delivery studies because fetuins are carrier proteins in the bloodstream [3] and asialofetuin-labeled liposomes was used for receptor-mediated transfer of DNA sequence to mouse liver cells [14]. Our study showed that several different forms of recombinant lectins specific to fetuin/asialofetuin could be designed modifying the number and array of tandem repeats. This versatility in structure may render recombinants of Rhodobindin useful in developing future drug-delivery system.

## 4. Experimental Section

### 4.1. Plant and Culture Conditions

Diploid plants of *A. callophyllidicola* were collected from Ochungdo, western coast of Korea and maintained in IMR medium [13]. Plants were grown at 15 °C in 16:8 h light and dark cycles with >20 μmol photons m^−2^·s^−1^ light provided by cool-white fluorescent bulbs [12].

### 4.2. Cloning and Design of Recombinant Lectins

Fresh female plants were ground to fine powder with a mortar and pestle in liquid nitrogen. Isolation of total RNA was performed using the RNeasy plant mini kit (Qiagen, Valencia, CA, USA) according to manufacturer’s protocol. First strand cDNA was synthesized using AccuScript High Fidelity 1st strand cDNA synthesis kit (Agilent, Palo Alto, CA, USA). Total RNA (5 μg) was added to the reaction mixture and incubated at 42 °C for 1 h. Synthesized cDNA was purified using PCR purification kit (Intron biotechnology, Seoul, Korea) and used as a PCR template.

Primers were designed for full cDNA of rhodobindin and six fragmentary recombinants based on the tandem repeat sequences of the primary structures (Table 1, Figure 1). The cDNAs were amplified with 2 μL of 1st strand cDNA in a total volume of 50 μL reaction mixtures containing 10 pmole of each primer. PCR was performed under the following conditions: cDNA was denatured at 95 °C for 3 min followed by 35 cycles of amplification (95 °C for 30 s, 60 °C for 30 s, 72 °C for 1–2 min) followed by 10 min at 72 °C. Amplified PCR products were isolated using a Qiagen gel extraction kit.

The above mentioned recombinants were cloned into an expression vector, pET28a(+) which has a *N*-terminus T7-tag and two His-tags on both sides. The cDNA and pET28a(+) (Invitrogen, Carlsbad, CA, USA) were digested with two enzymes, *EcoR*I and *Xho*I, for 2 h at 37 °C and the product was purified using the Qiagen gel extraction kit. The digested cDNA was cloned into pET28a(+) by incubation at 4 °C overnight with 4 units of T4 DNA ligase. The plasmid was transformed into expression host BL21 (λDE3) (Invitrogen, Carlsbad, CA, USA) and spread on LB agar plates containing 25 μg/mL of kanamycin. The colonies which have lectin sequences were isolated and sub-cultured in 10 mL of LB-kanamycin medium.

### 4.3. Optimization of rRhodobindin Expression

The transformants were inoculated in 10 mL of LB medium containing kanamycin (25 μg/mL) and cultured for 16 h at 37 °C. This subculture was inoculated (1:100) in 100mL of LB medium and cultured at 37 °C until they reach OD 0.2–1.4. When a designated OD was reached, samples were collected 2 h after adding IPTG (0.5 mM). To identify expression efficiency under different temperatures, *E. coli* cells (OD: 0.7) were transferred to different temperature (20, 30, 37 °C) after adding various concentrations of IPTG (0, 0.25, 0.5, 1, 2, 5 mM). Aliquots (5 mL) were collected after incubated at different temperatures.

Both insoluble and soluble proteins from each cell cultures were extracted and analyzed to determine expression levels. Insoluble fractions were prepared by heating samples for 5 min at 90 °C after treatment in 1× SDS-PAGE buffer (0.2 mL/1 mL culture) to the cell precipitates. The samples were centrifuged at 20,000×g for 10 min after heating and the supernatants were directly used as total protein extracts. Soluble fractions were obtained by sonication of 5mL of cell cultures with 1 mL of extraction buffer (1× PBS, 10 mM imidazole, 1 mM PMSF, pH 7.2). Soluble fractions were collected by centrifugation at 20,000× *g* for 10 min. The expression efficiency was determined by SDS-PAGE and measuring lectin activity.

### 4.4. Purification of Recombinant Lectins

Subculture (10 mL) of each transformed strain was inoculated into 1 L kanamycin-LB medium. IPTG induction was performed for 2 h by adding 0.5 mM of IPTG when turbidity reached OD 0.6–0.8. Cultures were centrifuged at 5000× *g* for 20 min and the supernatant was removed. The cell pellet was washed with PBS (Phosphate buffered saline, pH 7.2) twice and resuspended in 50 mL of extraction buffer (1X PBS, 10 mM imidazole, 1 mM of PMSF, pH 7.2). Cell lysate was prepared by sonication, 10 times for 10 s at 200 W with a 30 s cooling period between each burst, using an ultra-sonic device (Fisher Sonic Dismembrator Model 300, Fisher Scientific, Pittsburgh, PA, USA). Lysate was kept on ice at all times. The supernatant was collected by centrifugation at 20,000× *g* for 30 min at 4 °C.

Crude extract of each recombinant lectin was directly applied to Ni-NTA affinity chromatography (Qiagen, Valencia, CA, USA) using Biologic LP chromatography system (Bio-Rad, Richmond, CA, USA). Columns were washed with 10 volumes of extraction buffer with a 1 mL/min flow-rate. Recombinant protein was eluted by step gradient of 5 volume of 100 mM imidazole, 5 volumes of 250 mM of imidazole and then 5 volume of 500 mM of imidazole in extraction buffer. Protein concentrations and hemagglutinating activities of each fraction were determined. Fractions which showed hemagglutinating activity and single bands were pooled and dialyzed against 1X PBS pH 7.2.

### 4.5. Hemagglutinating Activity Assay and Determination of Carbohydrate Specificity

For the investigation of hemagglutinating activity we followed protocols described by Han *et al.* [12]. Horse blood was purchased from HanilComed (HanilComed, Seongnam, Korea) and washed with PBS. A serial two-fold dilution of the crude extract was made in a final volume of 25 µL saline in microtiter plate wells, and 25 µL erythrocyte suspensions (4%) was added sequentially to each well. The minimum amount of lectin required for complete agglutination was defined as 1 hemagglutination unit (HU).

Carbohydrate specificity was determined by inhibition tests of hemagglutinating activity. *N*-acetyl-glucosamine, *N*-acetyl-galactosamine, l-fucose, d-galactose, d-glucose, d-mannose, d-fructose and lactose at 500 mM, or glycoprotein, fetuin and asialofetuin in concentrations 100 mg/mL were used for inhibition test. Serial two-fold dilutions of sugar samples were prepared in PBS and mixed with an equal volume of 4 HU rRhodobindin. The mixture was mixed with equal volume (25 µL) of a 4% horse erythrocytes suspension. The minimum inhibitory concentration of the sugar in the final reaction mixture was calculated.

### 4.6. Effect of Temperature and Divalent Cations on the Agglutinating Activity of rRhodobindin

Heat stability was examined according to the method of Han *et al.* [32]. Heating aliquots of the purified rRhodobindine were prepared by incubation at various temperatures (4–100 °C) for 30 min. The heated lectin was cooled to room temperature and centrifuged at 12,000× *g* for 3 min to remove precipitate. The results were expressed by calculating the percentage of hemagglutinating activity shown by the heated samples compared with the non-heated sample (control) representing 100%. The effect of divalent metal ions was determined by dialysis to a metal-free buffer or by adding 5 mM of EDTA to the protein solution.

### 4.7. Mass Spectrometry

Protein bands on SDS-PAGE were excised and sent to Peptide Library Support Facility in POSTECH (Pohang, Korea) to analyze peptide sequence. The isolated proteins were in-gel digested with trypsin and cleaned with Zip-Tip (Millipore, Billerica, MA, USA). The peptide masses were determined using a matrix-assisted laser desorption ionization time-of-flight (MALDI-TOF) mass spectrometry (Applied Bio-system 4700 proteomic analyzer, Framingham, MA, USA) and peptides sequences were obtained using MALDI-MS/MS sequencing based on the manufacturer’s method.

## 5. Conclusions

Three functional recombinant lectins from marine red alga, *A. callophyllidicola* were developed using *E. coli* expression system; one from full cDNA sequence and two from fragmentary sequences of Rhodobindin. Carbohydrate-binding affinity of recombinant lectins were enhanced by an array of tandem repeats of the binding sites. The optimized process developed in this study was suitable for the quality-controlled production of high amounts of soluble recombinant lectins.

## Supplementary Files

Supplementary File 1
