# Supplementary material for: Functional Recombinants Designed from a Fetuin/Asialofetuin-Specific Marine Algal Lectin, Rhodobindin
Source: Mar Drugs. 2015 Apr 13;13(4):2183–95. doi: 10.3390/md13042183 (PMC4413206; doi:10.3390/md13042183)
Supplement: Supplementary File 1 [file marinedrugs-13-02183-s001.pdf]

Supplementary Information

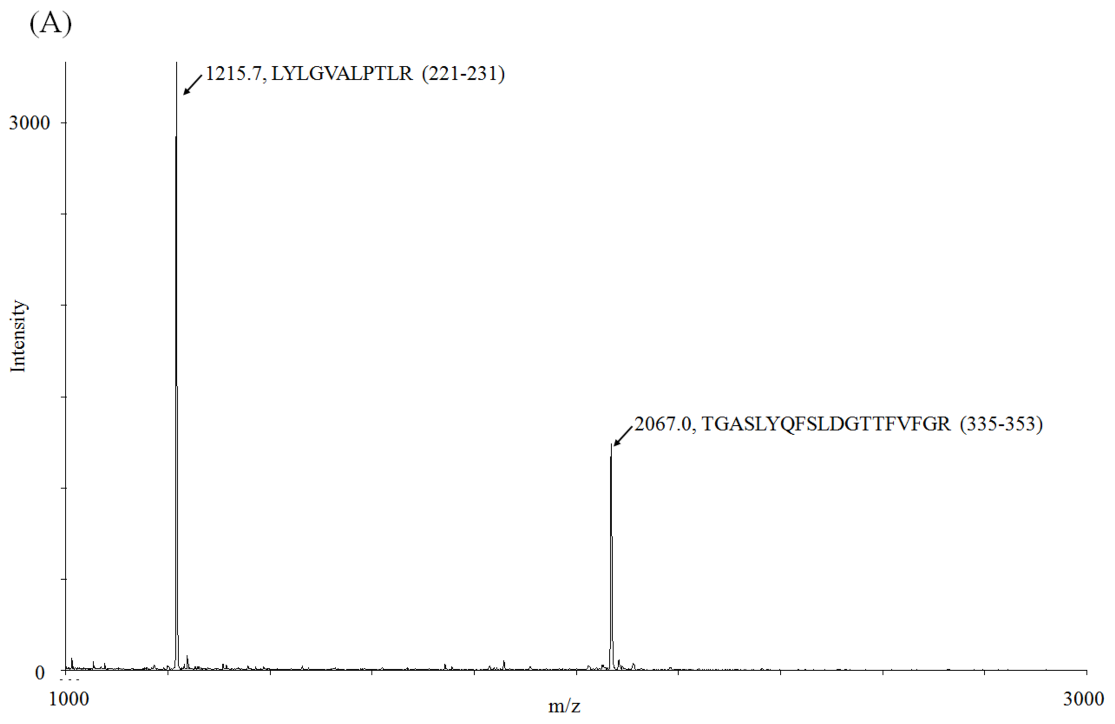

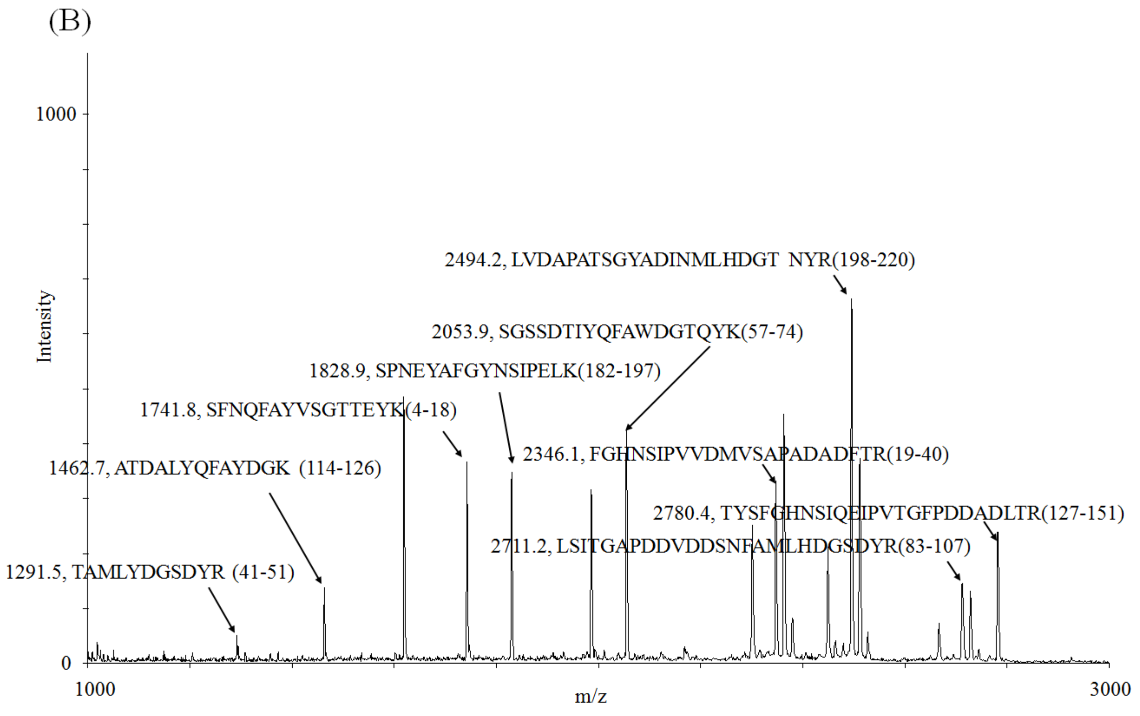

Figure S1. *Cont.*

(C)

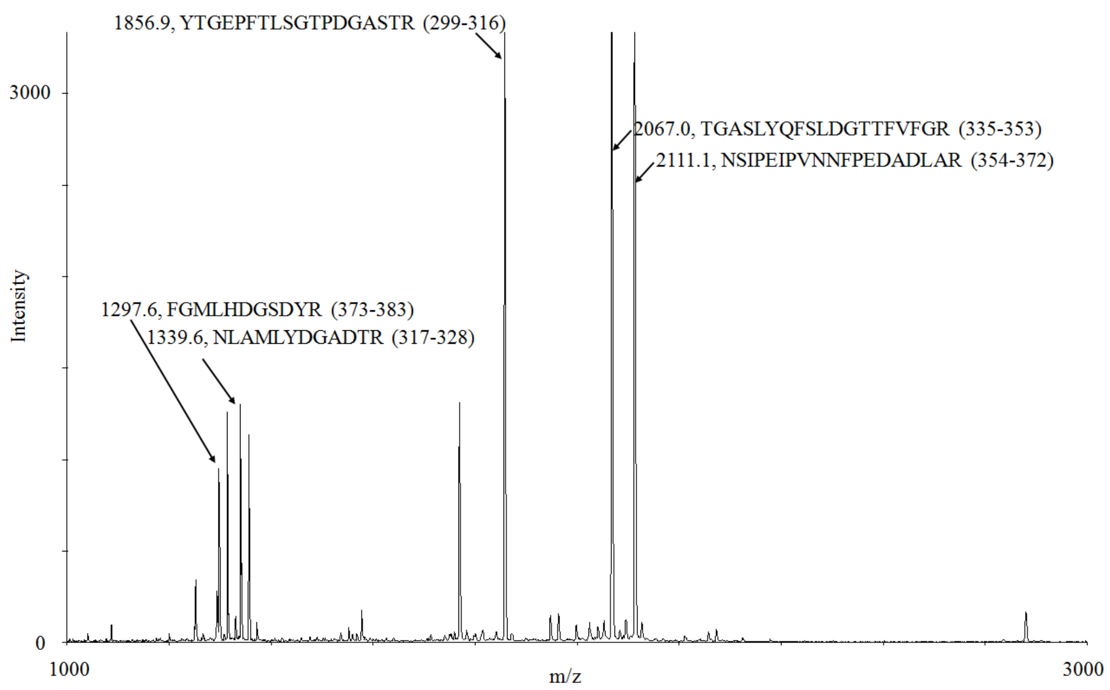

Figure S1. MALDI-TOF spectrometry. Arrows indicate matched peptide with predicted sequences (**A**) Whole protein; (**B**) rD1; (**C**) rD2.

```
         10        20        30        40        50        60
....|....|....|....|....|....|....|....|....|....|....|....|
MSRSFNQFAYVSGTTEYKFGHNSIPVVDMVSAPADADFTRTAMLYDGSDYRFYAFRSGSS
                                                   rD3
         70        80        90       100       110       120
....|....|....|....|....|....|....|....|....|....|....|....|
DTIYQFAWDGTQYKYGHNSIAKLSITGAPDDVDDSNFAMLHDGSDYRLYMRKRATDALYQ
              rD3
        130       140       150       160       170       180
....|....|....|....|....|....|....|....|....|....|....|....|
FAYDGKTYSFGHNSIQEIPVTGFPDDADLTRFDMLHDGSDYRYYAPSKTDVSKLYQAAFK
           rD1 (rD3+rD4)                         rD4
        190       200       210       220       230       240
....|....|....|....|....|....|....|....|....|....|....|....|
RSPNEYAFGYNSIPELKLVDAPATSGYADINMLHDGTNYRLYLGVALPTLRQFGFNRSTS
              rD4
        250       260       270       280       290       300
....|....|....|....|....|....|....|....|....|....|....|....|
AYQYGHDSIPEISLVDLPADTDWLRTAMCYDGQYQWFAFKAGSSDTLYKAEWNGTAYKYT
                                               rD5
        310       320       330       340       350       360
....|....|....|....|....|....|....|....|....|....|....|....|
GEPFTLSGTPDGASTRNLAMLYDGADTRLYLMSRTGASLYQFSLDGTTFVFGRNSIPEIP
            rD5                                 rD2 (rD5+rD6)
        370       380       390       400       410       420
....|....|....|....|....|....|....|....|....|....|....|....|
VNNFPEDADLARFGMLHDGSDYRFYALSRSESYKIYQGAFKRSPDEYLFGHNSIQSMPIT
                                        rD6
        430       440       450
....|....|....|....|....|....|
DAPEGTPTFDFNMLHDGSTYRLYLPIQAVA
          rD6
```

Figure S2. Amino acid sequence of Rhodobindin. Each domain was underlined (rD1–rD6).

© 2015 by the authors; licensee MDPI, Basel, Switzerland. This article is an open access article distributed under the terms and conditions of the Creative Commons Attribution license (http://creativecommons.org/licenses/by/4.0/).
